# Exercise alters LPS-induced glial activation in the mouse brain

**DOI:** 10.1042/NS20200003

**Published:** 2020-12-02

**Authors:** Bibiana C. Mota, Áine M. Kelly

**Affiliations:** Department of Physiology, School of Medicine and Trinity College Institute of Neuroscience and Trinity Biomedical Sciences Institute, Trinity College Dublin, University of Dublin, Dublin, Ireland

**Keywords:** astrocytes, Exercise, microglia, neuroinflammation

## Abstract

Experimental and epidemiological evidence suggest that modifiable lifestyle factors, including physical exercise, can build structural and cognitive reserve in the brain, increasing resilience to injury and insult. Accordingly, exercise can reduce the increased expression of proinflammatory cytokines in the brain associated with ageing or experimentally induced neuroinflammation. However, the cellular mechanisms by which exercise exerts this effect are unknown, including the effects of exercise on classic or alternative activation of astrocytes and microglia. In the present study, we assess the effects of nine consecutive days of treadmill running on the glial cell response to a single systemic injection of lipopolysaccharide (LPS) and, in parallel, the effects on spatial learning and memory. We show that prior exercise protects against LPS-induced impairment of performance in the object displacement task concomitant with attenuation of IL-1β, TNFα and IL-10 mRNA expression in the hippocampus. Assessment of isolated astrocytes and microglia revealed that LPS induced a proinflammatory response in these cells that was not observed in cells prepared from the brains of mice who had undergone prior exercise. The results suggest that exercise modulates neuroinflammation by reducing the proinflammatory microglial response, suggesting a mechanism by which exercise may be neuroprotective.

## Introduction

Resident glial cells play key roles in neuroinflammation. When activated, microglial cells can assume a proinflammatory state [1,2], characterised by increased expression of the proinflammatory cytokines interleukin-1β (IL-1β), interleukin-6 (IL-6) and tumor necrosis factor-α (TNF-α) [3,4]. However, they can also assume an anti-inflammatory state typified by production of anti-inflammatory cytokines, up-regulated expression of neurotrophic factors [5,6] and increased expression of Arginase-1 (Arg1), chitinase-like 3 (Ym1) and the mannose receptor Mrc-1, proteins associated with repair of inflammation-induced damage [7–9]. In macrophages, these states are termed M1 and M2 respectively, and while this nomenclature has featured in the literature with respect to microglia, it is likely that a variety of microglial phenotypes exist, especially *in vivo* [10].

An increasing body of evidence shows that astrocytes also contribute to inflammatory processes [11], demonstrated by the inflammation-associated increase in expression of glial fibrillary acidic protein (GFAP) in the brains of aged rodents and humans [12–15] and the ability of activated astrocytes to secrete cytokines [16]. Most recently, it has been suggested that, analogous to microglia, astrocytes can assume a pro- or anti-inflammatory phenotype (termed A1 or A2 [17]). The presence of pro-nflammatory glial cells is tightly linked with altered brain plasticity and cognitive decline [18,19]. For example, neuroinflammation negatively affects hippocampal neurogenesis, dendrite remodelling and long-term potentiation and these changes are associated with impaired cognitive function in aged rodents [20,21]. Systemic inflammation can trigger neuroinflammation and glial cell activation [22,23]; accordingly, systemic administration of lipopolysaccharide (LPS) has been widely used as a model of neuroinflammation, glial cell activation and cognitive impairment [24–26].

Exposure to anti-inflammatory cytokines *in vitro* can shift microglia from an M1 towards an M2 phenotype and mediate resolution of inflammation [27–29], suggesting that strategies that promote an anti-inflammatory state *in vivo* may be of potential neurotherapeutic value. Physical exercise is one such strategy, and many studies have demonstrated the potent cognitive enhancing and protecting effects of physical exercise in the young and aged brain and in several neurodegenerative diseases [30–33]. With regard to potential underlying mechanisms, exercise up-regulates expression of the anti-inflammatory cytokine IL-10 and attenuates the increase in expression of proinflammatory markers in the brain of aged rats [34], and can also attenuate LPS-induced proinflammatory cytokine expression [35]. Efforts to pinpoint the cells responsible for mediating such changes in the microenvironment of the central nervous system have revealed that both forced and voluntary exercise can modulate microglia and astrocyte activation in rodents [36–39] which, in some studies where cognitive function was assessed, was associated with memory enhancement [40].

Although physical activity clearly exerts modulatory effects on neuroinflammation, the ability of exercise to directly influence the brain microenvironment via activation of astrocytes and microglia towards pro- or anti-inflammatory phenotypes is not yet well understood. We have shown recently that a brief period of exercise improves cognitive function in aged animals via normalization of metabolic profile and function of microglia [41]. Here, we investigate whether, in mice, prior short-term exercise can prevent cognitive impairment induced by LPS-induced inflammation and have examined populations of microglia and astrocytes isolated from the brains of these mice for expression of markers reported to characterize the pro- and anti-inflammatory states.

## Materials and methods

### Animals

Six-month-old male C57BL/6 mice (20–30g, *n*=47) obtained from the Comparative Medicine Unit, Trinity College Dublin were used in these experiments. Mice were acclimated to the facility for 4 weeks and were handled daily for 5 min during this period. All experiments were performed under license in the Comparative Medicine Unit, Trinity College Dublin, in accordance with National and European directives on the protection of animals and were approved by the Animal Research Ethics Committee, Trinity College Dublin. Animals were group-housed, 4 to 5 per cage, under a 12:12-h light–dark cycle with food and water available *ad libitum* and with controlled temperature (22°C ± 2°C) and humidity (55% ± 5%).

### Experimental design

**Figure 1:**
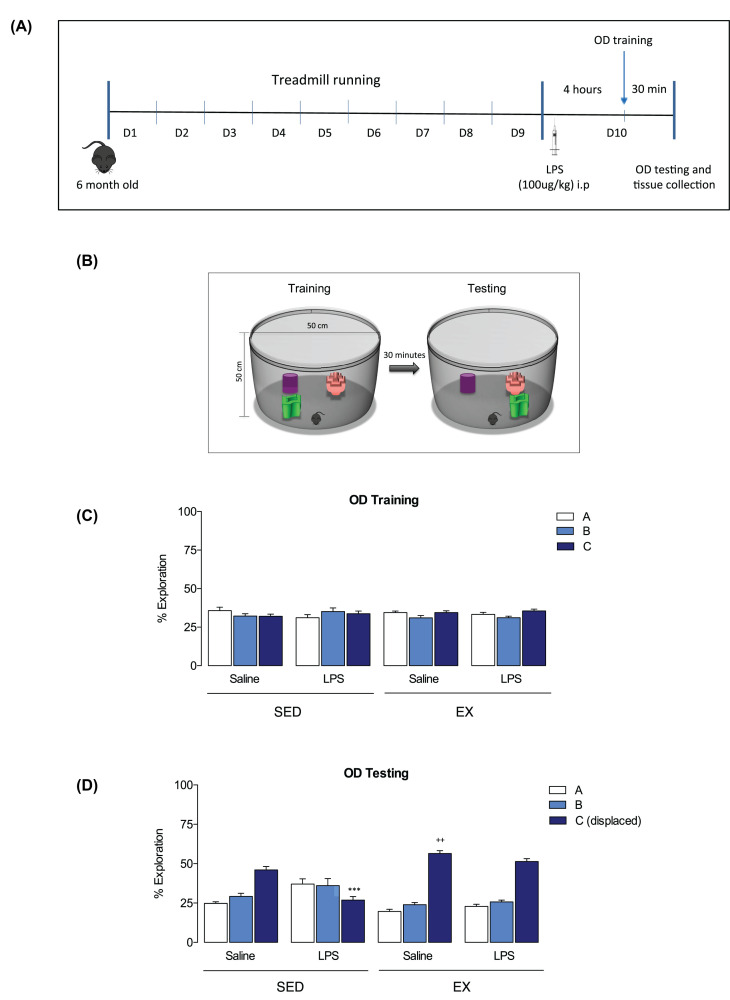
Effects of LPS and exercise on spatial learning and memory Experimental timeline of treadmill running protocol, lipopolysaccharide (LPS) injection and behavioural testing. (**A**) The object displacement (OD) task, with three different objects (A, B and C, where C is the displaced object in the testing phase), was used to test spatial learning and memory. (**B**) There was no preferential exploration of any object during the training phase by any group. (**C**) During the testing phase, exploration of Object C by the SED-LPS group was significantly decreased when compared with the other groups. (**D**) Exploration of Object C by the EX-SAL group was significantly enhanced when compared with SED-SAL, indicating an effect of exercise. All data are expressed as mean ± SEM, *n*=11–12 per group (two-way ANOVA with Bonferroni *post hoc* analysis, ***P<0.001, Object C exploration SED-LPS vs. other groups; ++*P*<0.01, exploration of Object C by EX-SAL when compared with SED-SAL).

Mice were randomly assigned to either sedentary (SED, *n*=23) or exercise (EX, *n*=24) group. EX mice experienced 1 h per day of moderate intensity exercise on a motorised treadmill for a period of nine consecutive days (Figure 1A). Twenty-four hours after the last session of exercise (Figure 1A), EX and SED mice received a single intraperitoneal (i.p.) injection of saline (0.9% NaCl (v/v)) or a sub-septic dose of lipopolysaccharide (LPS; *Escherichia coli* (100 μg/kg)). Four hours later, mice were trained in the object displacement (OD) task and tested 30 min after training. Mice were killed immediately following behavioural testing by sodium pentobarbital overdose and transcardial perfusion with sterile phosphate-buffered saline (PBS).

### Exercise protocol

Mice were familiarised to motorised rodent treadmills (Exer 3/6 treadmill, Columbus Instruments) daily for 5 days prior to the commencement of the exercise protocol by walking for 15–20 min at a belt speed of 4–6 m/min. The exercise protocol consisted of nine consecutive days of running for 1 h/day (belt speed 8–14 m/min, at zero inclination). Gentle hand prodding was used during the training phase to motivate animals to run. SED mice were placed on stationary treadmills for the same duration, to control for the effects of handling and exposure to a new environment.

### Object displacement task

Spatial memory was assessed using the object displacement task as previously described [42]. Briefly, mice were individually habituated to the open field in the absence of objects for 5 min for two consecutive days prior to training. The apparatus consisted of a black circular open field (diameter, 0.5 m; height, 0.48 m), placed in a dimly-lit room. Spatial cues were fixed to the wall of the open field. Objects were constructed from plastic toy blocks and were fixed to the floor of the open field, 10 cm from the walls. Four hours following Saline or LPS injection, mice were trained in the OD task (Figure 1B). During training, animals were placed individually in the open field with three different objects (Objects A, B and C) and were allowed to explore the objects for 5 min. Thirty minutes after training, 1 of the 3 objects (Object C) was displaced to a different quadrant of the open field and animals were allowed to explore for 5 min. Exploration was strictly classed as active; the mice had to be touching the object with at least their noses. Objects were cleaned with 70% ethanol between mice, to ensure the absence of olfactory cues. The time spent exploring each object was recorded during training and testing and was calculated as a percentage of the total exploration time.

### Tissue preparation and isolation of microglia and astrocytes

Mice were perfused intracardially with sterile ice-cold PBS. After perfusion, hippocampus was dissected free and the remaining brain tissue (except cerebellum) was placed in 1× Hank’s Balanced Salt Solution (HBSS; Invitrogen, U.K.), cross-chopped and homogenised for the isolation of an enriched population of microglia and astrocytes. Microglia and astrocytes were isolated from adult mice using previously described methods (Minogue et al, 2014). Briefly, cell suspensions were filtered through a cell strainer (70 μM), and cells were pelleted by centrifugation. Pellets were re-suspended in 75% Percoll (10 ml), overlaid with 25% Percoll (10 ml) and 1× PBS (6 ml), and centrifuged at 800 × ***g*** for 30 min at 4°C. After centrifugation, the myelin layer was carefully removed and an enriched microglial population was collected from the 25–75% interface (de Hass et al, 2007). In order to obtain an enriched astrocyte population, after the microglia layer was removed, cell suspensions were re-suspended in OptiPrep™ Density Gradient Medium (10 ml; Sigma-Aldrich, UK) and centrifuged once more at 800 × ***g*** for 30 min at 4°C. Remaining myelin was discarded and the enriched astrocyte population was collected.

### Real-time polymerase chain reaction (RT-PCR)

RNA was isolated from hippocampus, astrocytes and microglial cells using a Nucleospin RNAII kit (Macherey-Nagel, Germany). Samples were reverse transcribed into complementary DNA (cDNA) using the ABI High Capacity cDNA archive kit (Applied Biosystems, U.K.), following manufacturer’s instructions. Gene expression of targets was assessed using “Taqman gene expression assays” (Applied Biosystems), containing forward and reverse primers and a FAM-labeled MGB Taqman probe for each gene (Applied Biosystems). Probe IDs for the genes assessed were the following: β-actin (4352341E), arginase-1 (Mm00475988_m1), CD11B (Mm00434455_m1), GFAP (Mm01253033_m1), IBA-1 (Mm00479862_g1), IL-1β (Mm00434228_m1), IL-4 (Mm00445259_m1), IL-6 (Mm00446190_m1), IL-10 (Mm01288386_m1), iNOS (Mm00440502_m1), MRC1 (Mm00485148_m1), TNF-α (Mm00443258_m1) and Ym1 (Mm00657889_mH). RT-PCR was performed using Step One Plus TM Software (Applied Biosystems) and data were normalised to the expression of β-actin. RNA expression levels were quantified using the ΔΔCT method and presented as relative quotient (RQ) values.

### Statistical analysis

Data are presented as mean ± standard error of the mean (SEM). All data were analysed using two-way analysis of variance (two-way ANOVA) with Bonferroni post-hoc tests (GraphPad Prism software). Significance was accepted at *P*<0.05.

## Results

### Exercise protects against LPS-induced impairment in object displacement learning

The effect of injection of LPS on spatial learning and memory, with or without prior exercise, was assessed using the OD task (Figure 1B). No difference in the total time spent exploring objects during the training and testing phases was observed (data not shown), indicating no effect of exercise or LPS on general exploratory behaviour. During the training phase, all groups showed similar mean exploration time of all three objects, indicating no preference for any single object (Figure 1C; two-way ANOVA; no effect of treatment (F_3, 129_ = 8.989e-008) or object (F_2, 129_ = 1.107). During testing, object C was displaced to a different position in the open field, and exploration of object C was compared between groups. Exploration of object C by the SED-LPS group was significantly different to that of the other groups (*P*<0.0001) (Figure 1D; two-way ANOVA with Bonferroni *post-hoc*; effect of object F_2,129_ = 84.98 *P*<0.0001). Moreover, exploration of object C by the EX-LPS group was not significantly different to either of the saline-treated groups, indicating that exercise counteracted the effect of LPS injection on spatial learning and memory (significant interaction between group and object F_6,129_ = 25.84, *P*<0.001). This analysis also revealed a significant increase in exploration of Object C by the EX_SAL group when compared with the SED_SAL groups, indicating exercise-induced enhancement of task performance independent of LPS treatment (*P*<0.01).

### Exercise attenuated the LPS-induced increase in cytokine expression in the hippocampus

**Figure 2:**
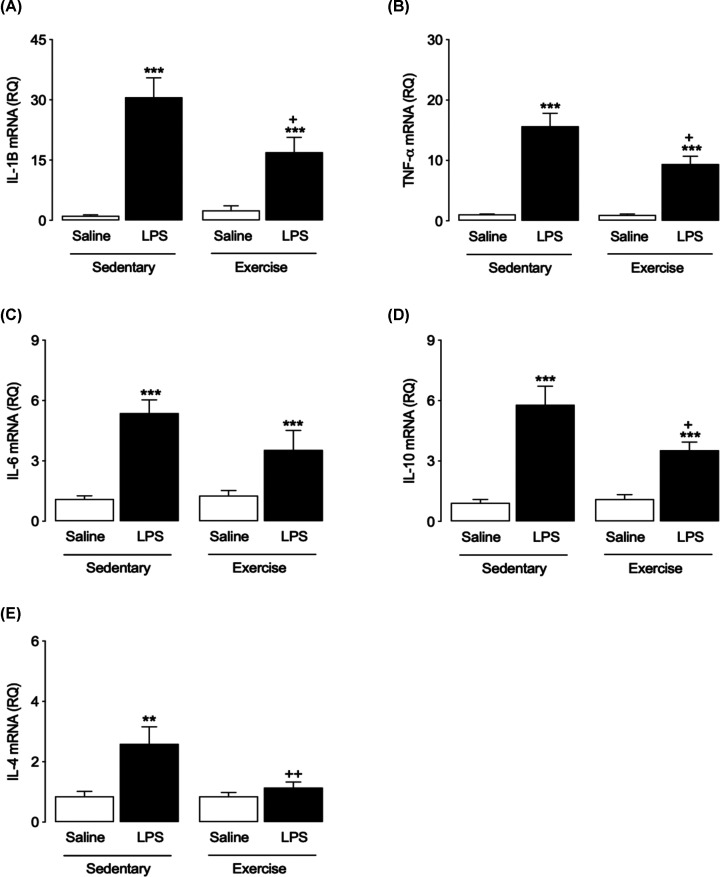
Effects of LPS and exercise on cytokine expression in the hippocampus Following LPS injection and behavioural testing, hippocampal mRNA expression of cytokines was assessed by RT-PCR. Expression of IL-1β and TNF-α was increased in SED and EX mice injected with LPS, but EX attenuated this increase (**A** and** B**). Exercise had no effect on the LPS-induced increase in expression of IL-6 mRNA. (**C**) The increase in IL-10 mRNA expression was down-regulated by exercise. (**D**) The LPS-induced increase in expression of IL-4 was completely blocked by exercise. (**E**) All data are presented as mean ± SEM, *n*=11–12 per group (two-way ANOVA with Bonferroni *post hoc* analysis, **P<0.01, ***P<0.001, Saline vs. LPS; ^+^*P*<0.05, ^++^*P*<0.01, SED-LPS vs. EX-LPS).

mRNA expression of pro- and anti-inflammatory cytokines in hippocampal tissue prepared from mice that had undergone behavioural testing was analysed by RT-PCR (Figure 2). Predictably, mRNA expression of the proinflammatory cytokines IL-1β TNF-α and IL-6 was increased in the hippocampus of SED-LPS mice (two-way ANOVA, *P*<0.0001, Figure 2A–C). The LPS-induced increase in IL-1β and TNF-α was attenuated in mice that had undergone prior exercise (*P*<0.05, Figure 2A,B), but this exercise effect was not observed in the case of IL-6 (Figure 2C). Similarly, LPS induced an increase in mRNA expression of the anti-inflammatory cytokines IL-10 (*P*<0.0001, Figure 2D) and IL-4 (*P*<0.05, Figure 2E) in the hippocampus of LPS-injected sedentary mice. Prior exercise attenuated the effect of LPS on IL-10 (*P*<0.05, Figure 1D) and completely blocked the effect of LPS on expression of IL-4 (*P*<0.05, Figure 2E).

### Exercise changes the expression of glial markers in the hippocampus following LPS challenge

**Figure 3:**
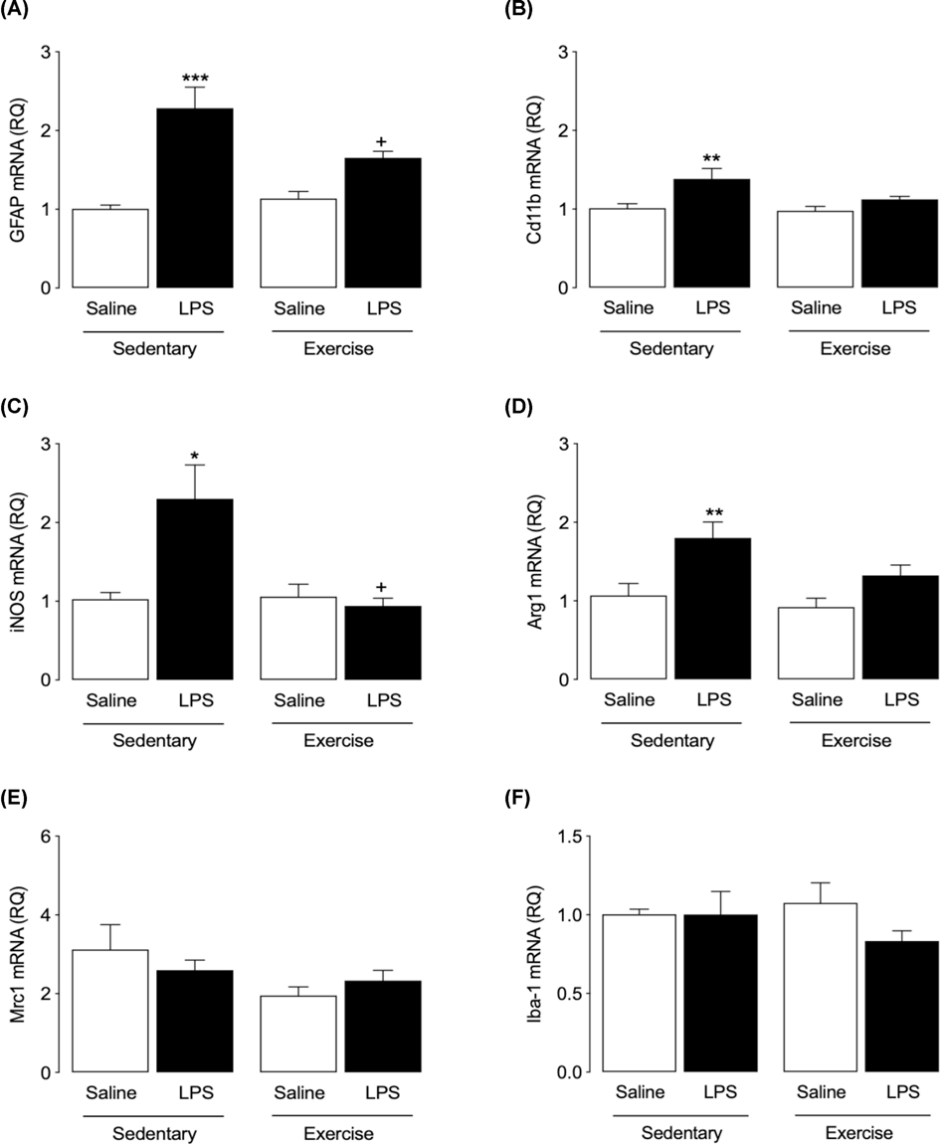
Effects of LPS and exercise on the expression of markers of glial cell activation in the hippocampus Changes in mRNA expression of markers of glial cell activation in hippocampus were measured using RT-PCR. A single LPS injection induced an increase in the mRNA expression of GFAP in SED mice that was significantly attenuated in EX mice (**A**). A significant increase in mRNA expression of CD11B, iNOS and Arg1 was observed in SED mice treated with LPS; this LPS-induced increase was not observed in the EX group. (**B**–**D**) Expression of neither MRC1 nor Iba-1 was affected by LPS or EX (**E** and** F**). All data are presented as mean ± SEM, *n*=11–12 per group (two-way ANOVA with Bonferroni *post hoc* analysis, *P<0.05, **P<0.01, ***P<0.001, Saline vs. LPS; ^+^*P*<0.05, SED-LPS vs. EX-LPS).

Having established the modulatory effect of prior exercise on LPS-induced changes in hippocampal cytokine expression, we investigated the effects of exercise on mRNA expression of glial cell activation markers in the hippocampus (Figure 3). GFAP mRNA expression was increased in SED-LPS mice (two-way ANOVA with Bonferroni *post-hoc, P*<0.0001, Figure 3A); this effect of LPS was significantly attenuated in the EX group (*P*=0.05, Figure 3A). CD11B mRNA expression was enhanced by LPS in the SED group only (Figure 3B). Similarly, LPS induced an increase in the expression of iNOS, typically classified as a phenotypic marker of M1 microglia, in the hippocampus of SED mice (*P*<0.05, Figure 3C), with the effect attenuated by exercise (*P*=0.05, Figure 3C). LPS also increased the expression of Arg1, classically a marker of M2 microglia, in the hippocampus of SED mice (*P*=0.0009, Figure 3D). However, expression of MRC1, also considered a M2 phenotype marker, was not affected by either LPS or EX (Figure 3E), while, no effect of LPS or EX was observed in the expression of Iba-1 (Figure 3F).

### Exercise changes expression of markers of microglial activation in an enriched population of microglia following LPS challenge

**Figure 4:**
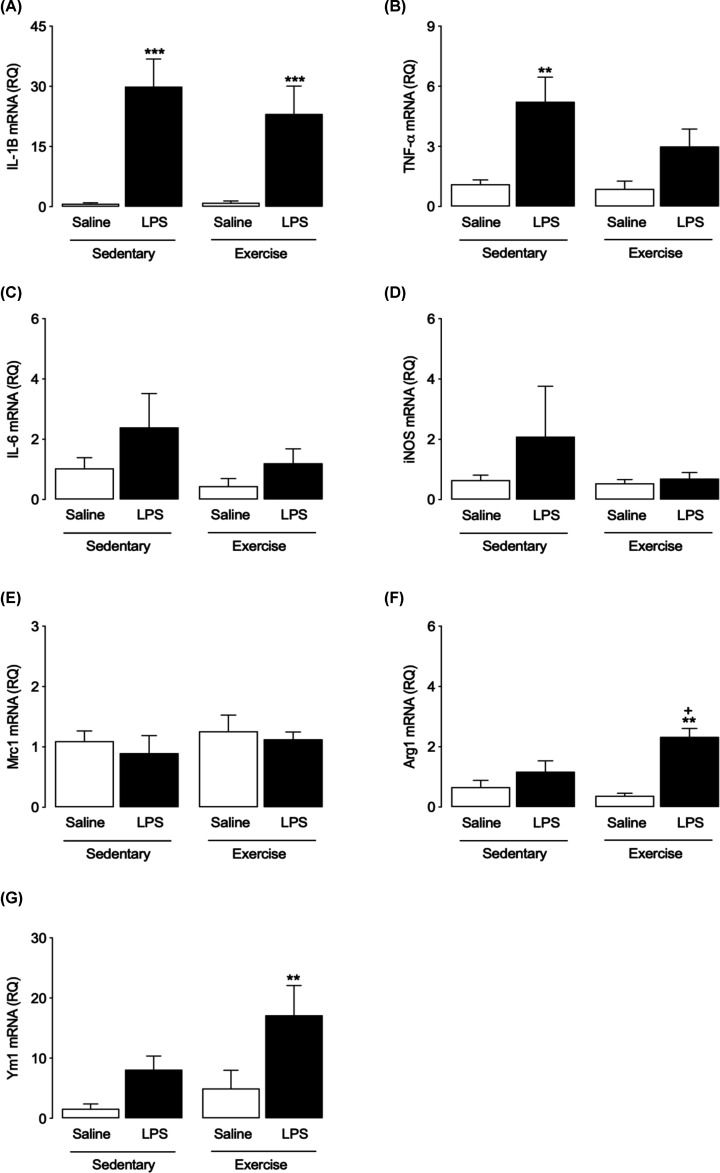
Effects of LPS and exercise on the expression of markers of microglial activation in an enriched population of microglia Expression of markers of M1-like and M2-like activation in a preparation of microglia isolated from brain tissue of SAL- and LPS-treated SED and EX mice was assessed by RT-PCR. Expression of IL-1β mRNA was increased in microglial cells in both SED and EX groups, following LPS injection (**A**) while LPS significantly increased the expression of TNF-α only in microglia prepared from SED mice (**B**) Neither LPS nor exercise affected the expression of IL-6, iNOS or MRC1 in any group (**C**–**E**). mRNA expression of Arg1 and Ym1 was significantly increased by LPS in microglia prepared from EX, but not SED, mice (**F** and** G**). All data are presented as mean ± SEM, *n*=5–6 per group (two-way ANOVA with Bonferroni *post hoc* analysis, **P<0.01, ***P<0.001, Saline vs. LPS; ^+^*P*<0.05, SED-LPS vs. EX-LPS).

Injection of LPS significantly increased mRNA expression of the proinflammatory cytokines IL-1β and TNF-α in microglia prepared from SED mice (two-way ANOVA with Bonferroni *post-hoc, P*<0.0001, *P*<0.05, respectively, Figure 4A,B), mimicking the change seen in hippocampus (Figure 2A,B). Expression of TNF-α was unaltered in microglia cells of EX mice (*P*=0.1886, Figure 4B). Although it appears that LPS may tend to increase mRNA expression of IL-6 and iNOS, statistical analysis showed that neither LPS nor exercise significantly altered their expression (two-way ANOVA with Bonferroni *post-hoc, P*=0.6468, *P*=0.4592, respectively, Figure 4C,D). mRNA expression of the M2 phenotype markers MRC1, Arg1 and Ym1, was also assessed in the microglial preparation. No change in mRNA expression of MRC1 was observed following LPS injection in either SED or EX groups (*p* = 0.4850, Figure 4E). However, LPS increased the expression of Arg1 and Ym1 only in microglia prepared from EX mice (*P*<0.05, Figure 4F,G) and the increase in mRNA expression of Arg1 was significantly greater in EX-LPS compared to SED-LPS mice (Figure 3F).

### Exercise did not alter the expression of markers of astrocyte activation in an enriched astrocyte population following LPS challenge

**Figure 5:**
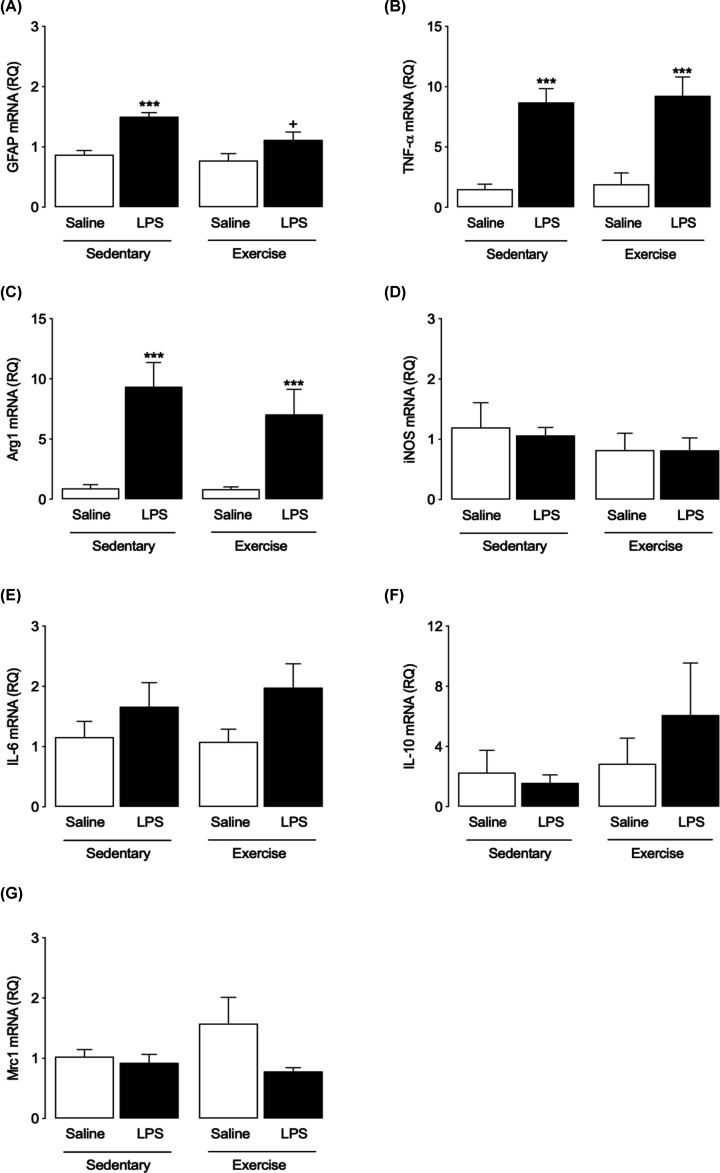
Effects of LPS and exercise on the expression of markers of astrocyte activation in an enriched astrocyte population Expression of markers of A1-like and A2-like activation astrocyte activation in a preparation of astrocytes isolated from brain tissue of SAL- and LPS-treated SED and EX mice was analysed using RT-PCR. Expression of GFAP mRNA was significantly increased by LPS in SED and EX groups, with the increase attenuated by EX. (**A**) Expression of TNF-α and Arg1 was up-regulated by LPS in both SED and EX groups (**B** and** C**). No significant difference in mRNA expression of iNOS, IL-6, IL-10 or MRC1 was observed between groups (**D**–**G**). All data are presented as mean ± SEM, *n*=5–6 per group (two-way ANOVA with Bonferroni *post hoc* analysis, ***P<0.001, Saline vs. LPS; ^+^*P*<0.05, SED-LPS vs. EX-LPS).

LPS induced a significant increase in the expression of GFAP mRNA in astrocytes prepared from SED mice (two-way ANOVA with Bonferroni *post-hoc, P*<0.0001, Figure 5A), which was reduced in EX mice (*P*<0.05, Figure 5A). However, mRNA expression of TNF-α was significantly increased by LPS to identical levels in both SED and EX groups (*P*<0.0001, Figure 5B). In contrast with results observed in microglia, mRNA expression of Arg1 in astrocytes was increased in both SED and EX groups (Figure 5C) with no effect of exercise observed. Conversely, neither LPS nor EX affected mRNA expression of iNOS and IL-6 in astrocytes (Figure 5D,E). Statistical analysis showed no significant effect of either LPS or EX on IL-10 mRNA (*P*=0.5448, *P*=0.2340, respectively, two-way ANOVA, Figure 5F). Neither did LPS or EX induce any change in mRNA expression of MRC1 in astrocytes (*P*=0.1168, *P*=0.4661, respectively, two-way ANOVA, Figure 5G), similar to results observed in hippocampus and microglia.

## Discussion

In the present study, we have observed that 9 days of treadmill exercise prevents acute LPS-induced impairment in spatial memory and some associated neuroinflammatory changes in both whole hippocampal tissue and in isolated populations of astrocytes and microglia. Systemic administration of LPS is well known to induce behavioural changes, collectively called ‘sickness behaviour’, characterised by decreased activity, neuroinflammation and cognitive impairment [43]. Although we show here that acute systemic administration of LPS induced increased cytokine expression in the hippocampus, this did not interfere with general exploratory and locomotor behaviour of mice during cognitive training or testing, since total object exploration did not differ between groups. LPS has specific effects on learning and memory independent of general malaise; for example, it interrupts consolidation of contextual fear memory [44], object recognition memory [45] and spatial learning in the water maze [46]. We extend these findings to demonstrate impairment of hippocampus-dependent short-term spatial memory in the object displacement task resulting from LPS injection. Furthermore, the cognitive-protecting effects of exercise observed in the present study support a large and robust literature. While treadmill exercise of as long as 5 weeks [47] or 6 weeks [48] and voluntary exercise of 9 weeks [35] attenuates neuroinflammation, improves cognitive performance and restores neurogenesis, here we show that as little as 9 days of exercise is sufficient to spare cognitive function in LPS-treated adult mice. Finally, the observation here that a short period of exercise significantly enhances spatial learning in saline-treated animals adds to the large volume of literature that reports the ability of exercise to act as a cognitive enhancer in healthy animals [49–51].

Systemic inflammation is characterised by increased concentrations of circulating cytokines that can impact the CNS, trigging a neuroinflammatory response and thereby affecting cognitive function [10,52,53]. Our finding that LPS induced a rapid (within 5 h) increase in mRNA expression of the proinflammatory cytokines IL-1β, TNF-α and IL-6 and of iNOS in the hippocampus agrees with previous reports [54,55]. This response was attenuated in exercised mice, suggesting a potent effect of exercise in minimising the central inflammatory response to systemic LPS administration and corroborating previous studies that have demonstrated exercise to counteract the increase of proinflammatory cytokines induced by bacterial toxins [56,57]. LPS is reported not only to up-regulate proinflammatory cytokines, but also to stimulate an early increase in the expression of anti-inflammatory cytokines such as IL-10 [58,59], suggesting this may be a mechanism to counterbalance the inflammatory response. Accordingly, our results demonstrate an LPS-induced increase in mRNA expression of IL-10 and IL-4 in the mouse hippocampus that was attenuated by prior exercise, reflecting the ability of exercise to suppress LPS-induced expression of proinflammatory cytokines.

Changes in cytokine expression in the brain secondary to systemic inflammation are coincident with activation of microglia and astrocytes [60,61]. We have assessed, in tissue prepared from whole hippocampus, a panel of markers that reflect activation states of glia, in an attempt to understand at the cellular level how exercise can modulate neuroinflammation. In parallel with increased cytokine expression in the hippocampus, we demonstrate significant induction of GFAP mRNA by LPS that was attenuated in exercised mice, suggesting a capability of prior exercise to dampen astrocyte activation. With respect to microglia, the LPS-induced increase in CD11b, iNOS and Arg1 mRNA expression was not observed in mice that had undergone prior exercise, while neither intervention affected expression of Iba1 or Mrc1. This allows us to suggest that prior exercise can reduce the response of microglia to systemic LPS challenge, albeit that differences in the pattern of expression of microglial markers were observed. This supports reports in the literature of a reduction in microglial activation in aged mice exposed to exercise [37,41]. Although Iba1 expression is reported to increase in activated microglia along with IL1β [62], the lack of change in Iba1 expression observed in these experiments does not necessarily indicate a lack of microglial activation induced by LPS in our study, which is suggested by the changes in expression of CD11b, since cytoskeletal rearrangements may not accurately represent an active inflammatory profile [63] and LPS has been shown to increase expression of cytokines in microglia independent of significant changes in microglial morphology [24]. Thus, assessment of the functional state of these cells, including cytokine secretion, is warranted in future experiments.

Overall, the results suggest a modulation of LPS-induced astrocytic and microglial activation in hippocampal tissue by exercise, but with differential expression of activation markers. To gain greater insight into the means by which these interventions may be modulating neuroinflammatory responses and hence altering cognitive function, expression of markers of glial activation was assessed in enriched populations of astrocytes and microglia isolated from whole brain. Our results show that the LPS-induced increase in IL-1β and TNFα mRNA expression observed in hippocampus was mimicked in isolated microglia, indicating activation of these cells, but that exercise prevented the increase in TNFα only. No significant change in expression of IL-6 or iNOS was seen. In parallel, we demonstrated that exercise can increase classical M2-like phenotype markers, such as Arg-1 and Ym1, in response to LPS. Thus, the major effect of exercise observed in this population of isolated cells was a modification of the balance between markers of classically activated and alternatively activated glia. This altered balance and its as yet unknown functional effects may be contributing to the exercise-induced reduction in inflammation observed in hippocampal tissue which could, in part, be the means by which exercise protects against the LPS-induced impairments in spatial memory observed. This contrasts with a recent study suggesting that 8 weeks of voluntary wheel running had minimal effects on expression of M2 markers such as Arg1, IL-1ra, TGF-β and CD206 in the hippocampus of adult and aged mice [64], although in that study animals were administered a combination of the anti-inflammatory cytokines IL-4/IL-13 in contrast with the systemic proinflammatory immune challenge presented here. In the case of the isolated astrocyte population, the reduction in LPS-induced GFAP mRNA expression by exercise reflects the results observed in whole hippocampal tissue, suggesting that exercise can reduce astrocytic activation. However, assessment of expression of inflammatory markers showed that the effects of LPS were identical in astrocytes isolated from both sedentary and exercised rats. Taken together, these results prompt us to suggest that exercise has more potent effects in microglia when compared with astrocytes. However, it must be acknowledged that our assessment is based on mRNA expression of these markers. In addition to the assessment of cell function suggested above, analysis of protein expression would allow a more complete picture of the activation state of the isolated cells. Furthermore, it must be noted that it may not be accurate to make direct comparisons between mRNA expression patterns in hippocampal tissue and in the isolated microglia and astrocyte populations. First, the changes observed in hippocampus may reflect expression of proteins in cells other than microglia and astrocytes, e.g. infiltrating immune cells or even neurons [65]. Second, the process of cell purification may have induced changes in cytokine mRNA expression. One further limitation of the study is that we have carried out our experiment in male mice only. Sex-specific differences in activation and function of microglia have been reported, though this remains an under-analysed phenomenon [66]. Future experiments should assess any sex-specific differences in the response of glia to exercise and inflammatory challenge.

Our data are consistent with reports of a general anti-inflammatory effect of exercise both peripherally and centrally [67,68]. With respect to inflammation, the majority of published studies have investigated modulation of microglial rather than astrocytic activation by exercise [69]. Furthermore, microglia also demonstrate a larger response to LPS stimulation when compared with astrocytes [63], which could at least partly explain the more modest effects of LPS and exercise in modulation of astrocytes observed in the present study when compared with microglia. These modulatory effects of exercise may underlie the rescued cognitive impairment observed in the present study.

We conclude that exercise can induce its protective effects on cognitive function via impeding the classically activated phenotype of microglia. It must be noted that the changes observed in these experiments are in expression of mRNA and not protein, nor have we assessed cell morphology in the brains of these animals. Here we focus on early changes (within 5 h) in cognitive function in response to a sub-septic dose of LPS, and concomitant changes in LPS-induced markers of inflammation, which are more likely within this timeframe to be captured by mRNA rather than protein expression within cells [22,45,55]. Accordingly, it is likely that the effects of inflammation on neuronal function in these mice are mediated by rapidly induced changes such as secretion of proinflammatory cytokines, which can decrease synaptic excitability and neuronal plasticity [70], and exercise functions to attenuate this response; this hypothesis could be addressed in future experiments. In summary, our results demonstrate prior short-term exercise to protect against LPS-induced spatial memory impairment, within 5 h of systemic LPS administration. The mechanisms mediating the effects of exercise are likely to involve the anti-inflammatory modulatory action of exercise, as we demonstrated that exercise attenuated central expression of inflammatory cytokines, and decreased expression of general inflammatory markers, particularly in microglia.

## Abbreviations

HBSS: Hank’s Balanced Salt Solution
iNOS: inducible nitric oxide synthase
TGF-β: tumor necrosis factor β
IL-1β: interleukin-1β
IL-6: interleukin-6
LPS: lipopolysaccharide
PBS: phosphate-buffered saline
RT-PCR: real-time polymerase chain reaction
TNF-α: tumor necrosis factor-α
